# MicroRNA-125b reverses oxaliplatin resistance in hepatocellular carcinoma by negatively regulating EVA1A mediated autophagy

**DOI:** 10.1038/s41419-018-0592-z

**Published:** 2018-05-10

**Authors:** Wei-Wei Ren, Dan-Dan Li, Xiaolan Chen, Xiao-Long Li, Ya-Ping He, Le-Hang Guo, Lin-Na Liu, Li-Ping Sun, Xiao-Ping Zhang

**Affiliations:** 1 Department of Medical Ultrasound, Shanghai Tenth People’s Hospital, Ultrasound Research and Educational Institute, Tongji University School of Medicine, Shanghai, 200072 China; 2 Department of Radiotherapy, Shanghai Tenth People’s Hospital, Ultrasound Research and Educational Institute, Tongji University School of Medicine, Shanghai, 200072 China; 3 Department of Interventional & Vascular Surgery, Tongji University School of Medicine, Shanghai, 200072 China

## Abstract

EVA1A (also known as transmembrane protein 166) is a transmembrane protein involved in the regulation of autophagy that acts as an adaptor protein to recruit or bind proteins in the lysosome or endoplasmic reticulum. In the present study, we identified EVA1A as a target of microRNA-125b (miR-125b), a member of a highly conserved family of miRNAs that has been proposed as a biomarker for hepatocellular carcinoma (HCC). Analysis of oxaliplatin-sensitive and oxaliplatin-resistant HCC cell lines showed that miR-125b is downregulated in resistant cells and its overexpression in sensitive cells decreased resistance to oxaliplatin by inhibiting cell proliferation, migration and epithelial–mesenchymal transition (EMT). EVA1A expression was shown to be upregulated in tissue samples from oxaliplatin-resistant HCC patients, and its ectopic expression partially induced autophagy and reversed the effect of miR-125b on inhibiting the growth of oxaliplatin-resistant cell lines and xenograft tumors. Taken together, our results suggest that miR-125b plays a role in the resistance of HCC cells to chemotherapy via a mechanism involving the downregulation of EVA1A-mediated autophagy.

## Introduction

Hepatocellular carcinoma (HCC) is a severe cancer with an increasing incidence worldwide, ranking fifth as the most common cancer and the third leading cause of cancer-related deaths in men^1,2^. The overall 5-year survival from HCC is less than 12%, and a three-fold increase in its incidence between 1975 and 2007 has made it the fastest rising cause of cancer-related death in the United States^3^. HCC is most often diagnosed late, at a time when curative treatments are not feasible; it is characterized by a low resectability rate, high recurrence after surgery, and poor response to treatment, making its prognosis grave and resulting in a serious burden for healthcare systems^2,4^. Currently, the only approved systemic therapy for the treatment of HCC is sorafenib, a tyrosine kinase inhibitor^5^. Oxaliplatin-based chemotherapy has recently been shown to be effective for the treatment of advanced HCC; however, the development of resistance to treatment severely limits its efficacy.

MicroRNAs (miRNAs) are small (10–22 nucleotides) endogenous single-stranded RNAs that regulate gene expression by binding to the 3′-untranslated region (UTR) of target genes, which leads to translational repression or degradation of the targeted transcript^6^. Aberrant expression of miRNAs has been implicated in the pathogenesis of several diseases including cancer, and miRNAs can function as tumor suppressors or oncogenes depending on their target genes. miR-125b is downregulated in numerous types of cancers including HCC, and ectopic expression of miR-125b inhibits proliferation, invasion and the tumorigenic potential of liver cancer cells, suggesting that it plays a tumor suppressor role in liver cancer^7–10^. miR-125b has been proposed as a biomarker to predict the prognosis of patients with HCC, however, its role in drug resistance remains unknown^11^.

Transmembrane protein 166 (TMEM166, also known as FAM176A or EVA1A) is a lysosomal and endoplasmic reticulum-associated protein that plays a role in programmed cell death and can facilitate both autophagy and apoptosis^12^. However, the function of EVA1A is unknown in oxaliplatin-resistant HCC.

In the present study, we examined the role of miR-125b in the resistance of HCC cells to oxaliplatin treatment and elucidated a potential mechanism by which miR-125b decreases oxaliplatin resistance in HCC via the downregulation of its target EVA1A.

## Results

### Differential expression of miR-125b in oxaliplatin-resistant and sensitive HCC cell lines

**Fig. 1:**
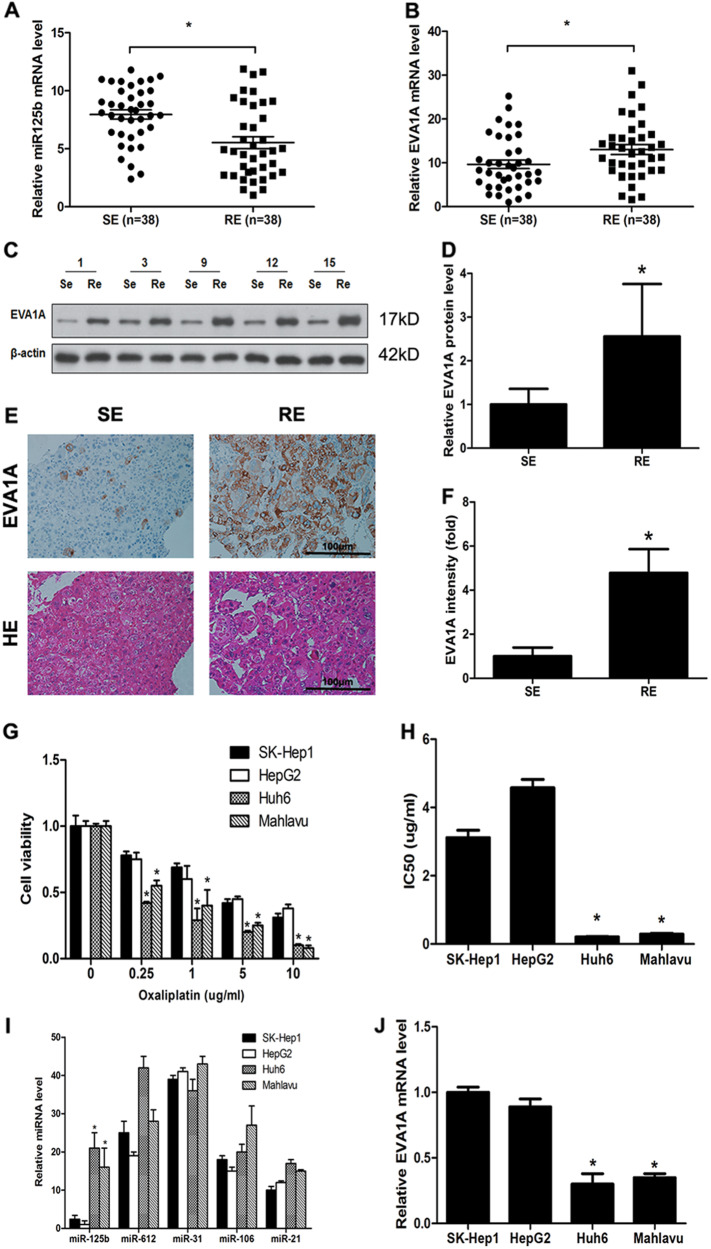
miR-125b and EVA1A expression in oxaliplatin-resistant and sensitive HCC cell lines. **a**, **b** miR-125b and EVA1A expression in oxaliplatin-resistant (*n* = 38) and non-resistant (*n* = 38) HCC patients’ tumor tissues analyzed by qRT-PCR. **c**, **d** EVA1A expression analyzed by western blotting in tumor tissues and quantified. **e**, **f** EVA1A expression analyzed by immunohistochemistry in tumor tissues and quantified. **g**, **h** Viability of oxaliplatin-resistant (SK-Hep1 and HepG2) and oxaliplatin-sensitive (Huh6 and Mahlavu) cell lines in response to different concentrations of oxaliplatin measured using the CCK-8 assay. **i** Relative levels of expression of miR-125b in oxaliplatin-resistant and sensitive HCC cell lines. **j** EVA1A expression levels in HCC cell lines determined by real-time PCR. *P < 0.05

The expression of miR-125b was determined by real-time PCR in tissues from oxaliplatin-resistant and oxaliplatin-sensitive HCC patients. Expression levels of miR-125b were lower in resistant tissues than in sensitive tissues (Fig. 1a). In contrast, EVA1A expression was higher in resistant tissues than in sensitive tissues when determined by real-time PCR, western blotting, and immunohistochemistry (Fig. 1b-f). Then, the correlations of miR-125b and EVA1A expression and special clinicopathological parameters and prognosis of HCC were analyzed, as shown in Table 1. The results showed that miR-125b and EVA1A expression were obviously associated with tumor size, tumor differentiation and distant metastasis stage.

**Table 1:** Relationship between miR-125b and EVA1A expression and the clinical pathological characteristics in HCC patients

Clinic pathological features		No. of cases	miR-125b (*n*, %)		*p-*value	EVA1A (*n*, %)		*P*-value
			Low	High		Low	High	
Gender	Male	48	21	27	>0.05	22	26	>0.05
	Female	28	12	16		13	15	
Age	≤45	23	11	12	>0.05	9	14	>0.05
	>45	53	24	29		21	32	
HBsAg	Negative	24	14	10	>0.05	11	13	>0.05
	Positive	52	29	23		24	28	
HBV infection	Absent	21	8	13	>0.05	5	16	>0.05
	Present	55	26	29		23	32	
Albumin(g/L)	≤35	34	21	13	>0.05	17	17	>0.05
	>35	42	29	13		20	22	
Serum AFP(μg/L)	≤400	40	17	23	>0.05	27	13	<0.05
	>400	36	21	15		12	24	
Liver cirrhosis	Absent	31	13	18	>0.05	16	15	>0.05
	Present	45	23	22		19	26	
Child-Pugh classification	A	47	19	28	>0.05	21	26	>0.05
	B	29	12	17		19	10	
Tumor size(cm)	≤5	30	7	23	<0.05	25	5	<0.05
	>5	46	32	14		15	31	
Tumor number	Single	51	24	27	>0.05	17	34	>0.05
	Multiple	25	11	14		13	12	
Tumor differentiation	I-II	41	17	24	<0.05	31	10	<0.05
	III-IV	35	29	6		11	24	
Distant metastasis stage	M0	42	13	29	<0.05	34	8	<0.05
	M1	34	27	7		5	29	
Prior chemotherapy	Absent	47	25	22	>0.05	19	28	>0.05
	Present	29	19	10		15	14	

Oxaliplatin-tolerant SK-Hep1 and HepG2 HCC cell lines and oxaliplatin-sensitive Huh6 and Mahlavu lines were treated with increasing concentrations of oxaliplatin and then cell viability was assessed using the CCK-8 method. Cell viability decreased in a dose-dependent manner in all cell lines, with a significantly greater decrease in viability in the Huh6 and Mahlavu cells than in the resistant SK-Hep1 and HepG2 cells (Fig. 1g). IC50 values confirmed these results (Fig. 1h). Microarray analysis of miRNA expression in the four HCC cell lines identified miR-125b as a differentially expressed miRNA, with significant downregulation in oxaliplatin-resistant SK-Hep1 and HepG2 cells (Table 2). This was confirmed by real-time PCR, which showed significantly lower miR-125b and higher EVA1A expression in resistant SK-Hep1 and HepG2 cells than in the sensitive Huh6 and Mahlavu cells (Figs. 1i, j).

**Table 2:** Downregulated miRNAs in the oxaliplatin-resistant HCCs

miRNA	Fold-change (SK-Hep1)	Fold-change (HepG2)
hsa-miR-4486	2.97E–06	0.00033
hsa-miR-125b	3.04E–06	9.33E–05
hsa-miR-3147	4.78E–06	1.33838
hsa-miR-181	7.79 E–05	1.71E–06
hsa-miR-26	8.80 E–05	0.00919
hsa-miR-4767	0.00921	0.00014
hsa-miR-3907	0.01163	1.00392
hsa-miR-574-3p	0.02519	0.46317
hsa-miR-5196-5p	0.02641	0.77422
hsa-miR-765	0.03321	5.85E–06

### miR-125b inhibits cell proliferation, invasion, and epithelial–mesenchymal transition (EMT) under the Oxaliplatin treatment

**Fig. 2:**
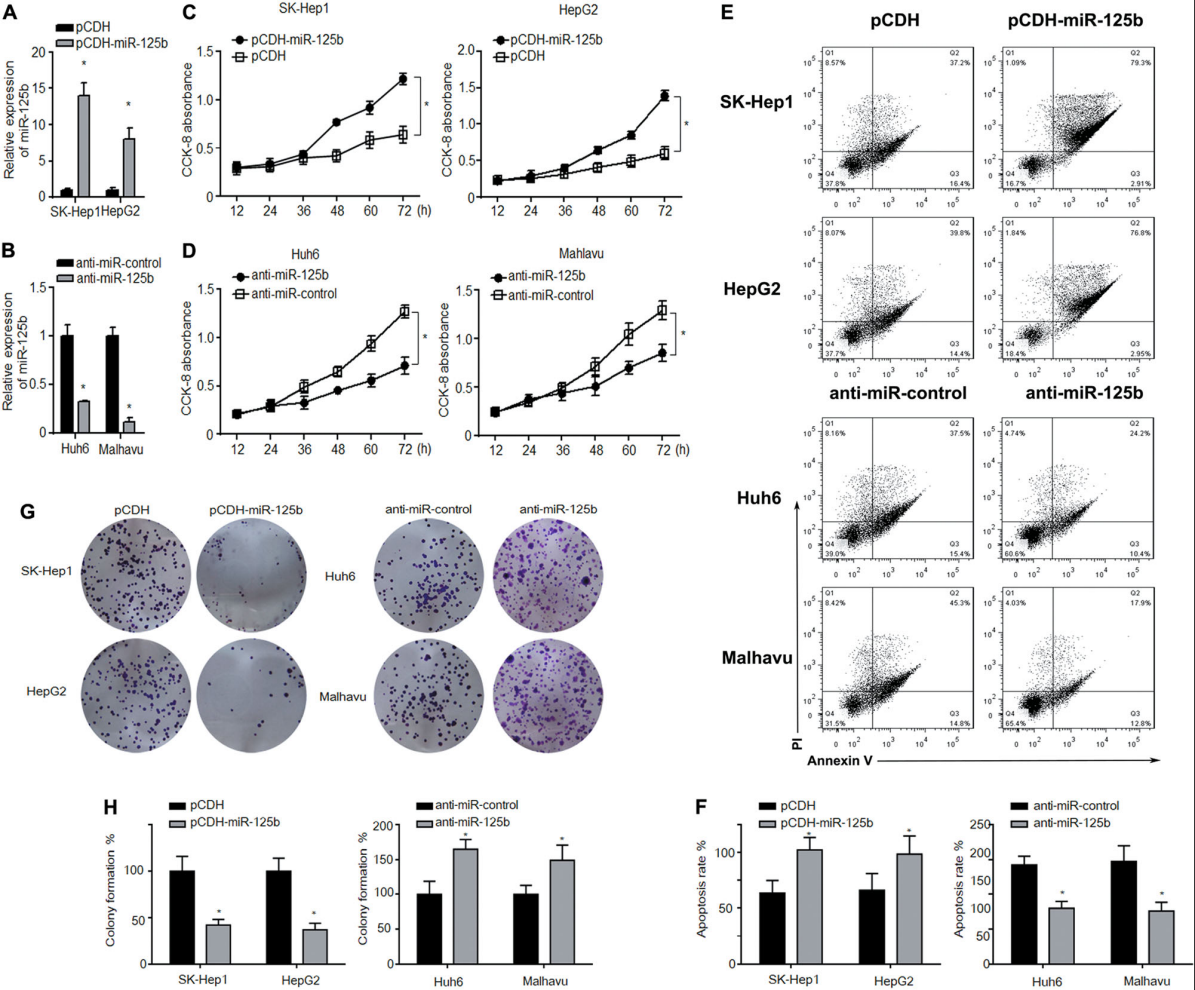
Effect of miR-125b on cell proliferation under the Oxaliplatin treatment. miR-125b was overexpressed in the oxaliplatin-resistant HCC cell lines SK-Hep1 and HepG2 (**a**, **b**) and inhibited in the oxaliplatin-sensitive cells Huh6 and Mahlavu (**c**, **d**). Cell proliferation was assessed by the CCK-8 method in response to oxaliplatin exposure for different times. **e**, **f** Analysis of apoptosis rates by flow cytometry and quantified after 72 h oxaliplatin treatment. **g**, **h** Colony number was assessed by Colony formation assays and quantified after 72 h oxaliplatin treatment. Data are expressed as means ± SEM (*n* = 3). *P < 0.05

**Fig. 3:**
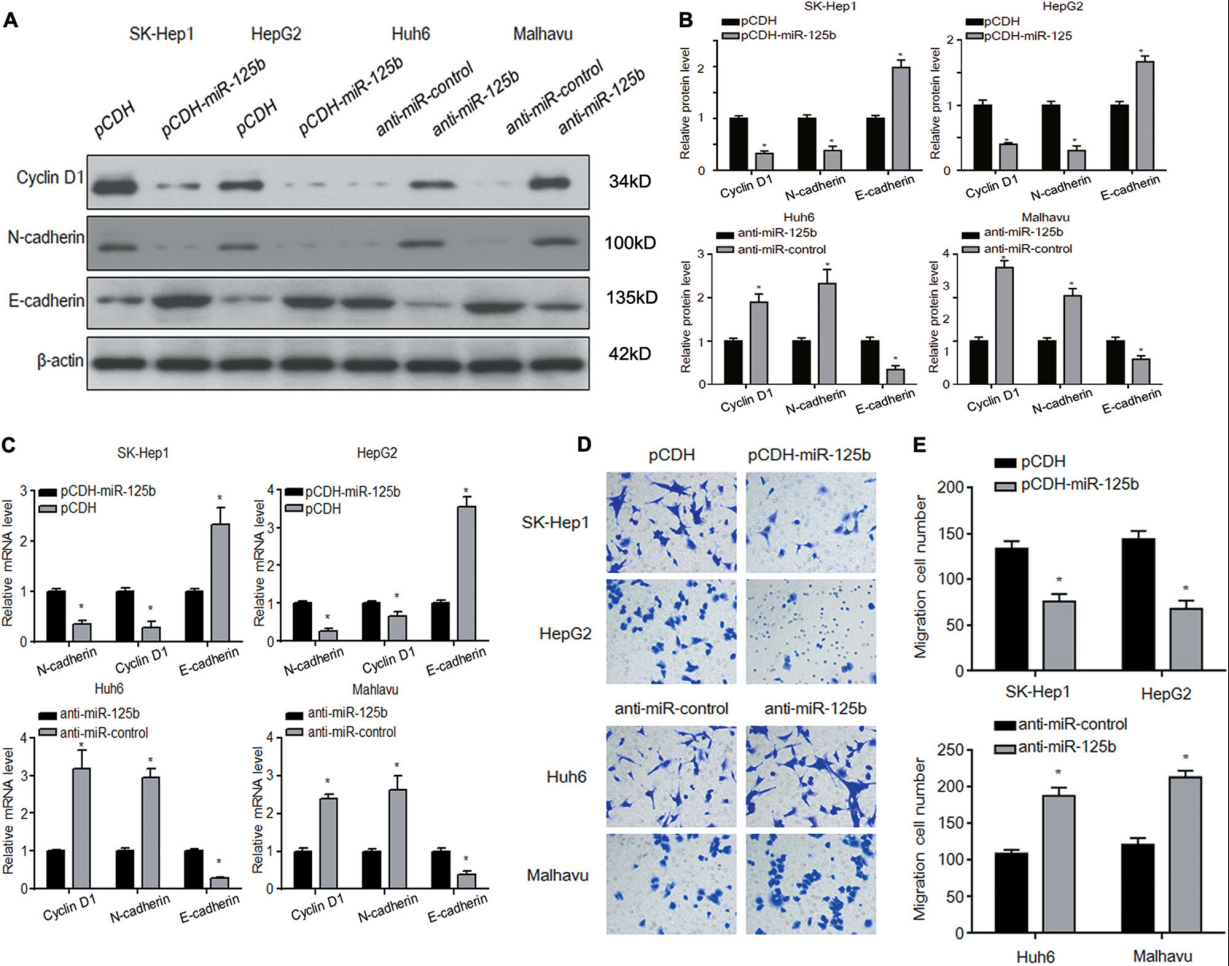
Effect of miR-125b on cell invasion and the expression of markers of epithelial–mesenchymal transition. **a**–**c** The expression of EMT markers was determined by western blot and real-time PCR and quantified. **d**, **e** Cell migration was assessed using Transwell assays and quantified. Data are expressed as means ± SEM (*n* = 3). *P < 0.05

To examine the role of miR-125b in HCC, miR-125b was overexpressed in oxaliplatin-resistant SK-Hep1 and HepG2 cells and inhibited in oxaliplatin-sensitive Huh6 and Mahlavu cells. Cell proliferation was assessed in cells treated with oxaliplatin (5 μg/ml) at a number of time points over 72 h. miR-125b overexpression decreased the cell proliferation of SK-Hep1 and HepG2, whereas treatment with anti-miR-125b decreased the proliferation of Huh6 and Mahlavu cells (Figs. 2a–d). Cell proliferation, apoptosis, and colony number were assessed in resistant and sensitive cell lines with miR-125b inhibited (Figs. 2e–g). Cell proliferation and colony formation were increased in sensitive cells whereas apoptosis was reduced. The opposite occurred in resistant cell lines. The expression of EMT markers were assessed in oxaliplatin-resistant and oxaliplatin-sensitive HCC cells. In resistant cells, miR-125b overexpression downregulated cyclin D1 and N-cadherin and upregulated E-cadherin at the mRNA and protein levels, indicating that it inhibited EMT. Conversely, miR-125b inhibition in sensitive cells upregulated cyclin D1 and N-cadherin and downregulated E-cadherin at the mRNA and protein levels, indicating that EMT was promoted in oxaliplatin-sensitive HCC cells (Figs. 3a–c). Cell migration was assessed using Transwell assays (Figs. 3d, e). When miR-125b was overexpressed, cell migration was reduced in oxaliplatin-resistant cells whereas inhibiting miR-125b expression increased migration in oxaliplatin-sensitive cells compared with cells transfected with a negative control vector. Taken together, these results indicate that miR-125b may reverse oxaliplatin resistance by suppressing cell proliferation and invasion.

### EVA1A is a target of miR-125b

**Fig. 4:**
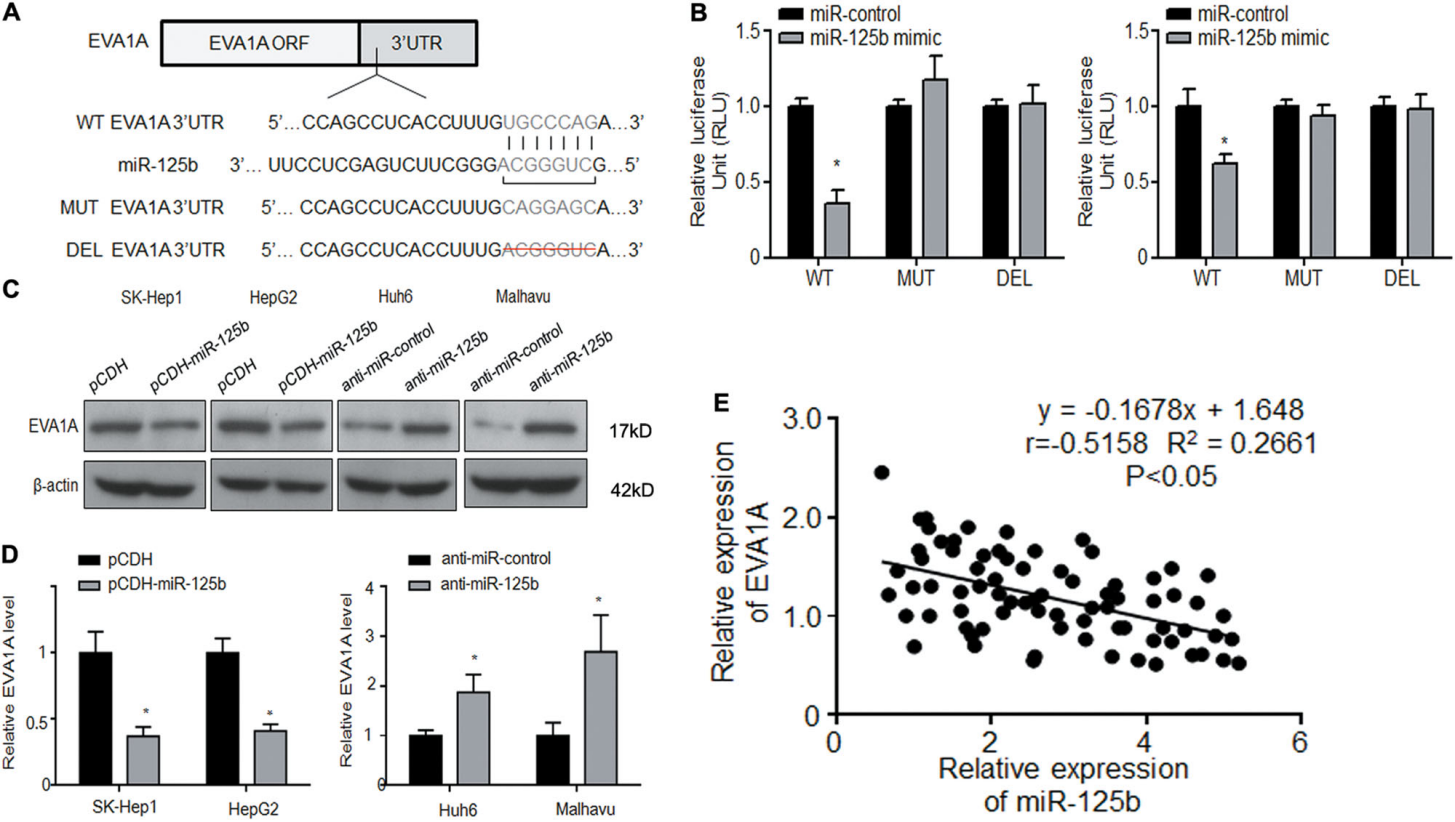
EVA1A is a target of miR-125b. **a** The wild-type (WT), mutated (MUT), or deleted (DEL) EVA1A 3′-UTR was transfected into HCC cells with or without synthetic miR-125b mimics. **b** Relative luciferase activity was determined using luciferase assays. **c**, **d** EVA1A expression was determined in oxaliplatin-sensitive and resistant HCC cells by western blotting and quantified. **e** The correlation between miR-125b and EVA1A expression in human HCC samples was determined by Spearman’s correlation analysis. Data are expressed as means ± SEM (*n* = 3). *P < 0.05

EVA1A was identified as a potential target of miR-125b. To confirm that EVA1A is a target of miR-125b, we generated luciferase reporter constructs containing the wild-type 3′-UTR of EVA1A or two constructs with a mutation or deletion in the putative miR-125b binding site in the 3′-UTR of EVA1A (Fig. 4a). The constructs were co-transfected into the four HCC cell lines together with miR-125b mimics or miR-control, and luciferase activity was determined using a luciferase assay. As shown in Figure 4b, miR-125b overexpression inhibited the activity of the wild-type but not that of the mutant or deleted EVA1A promoter, suggesting that miR-125b interacts with the EVA1A 3′-UTR and inhibits its promoter activity. Western blot assessment of the expression of EVA1A in HCC cells treated with pCDH-miR-125b or anti-miR-125b or their respective controls showed that EVA1A expression was higher in oxaliplatin-resistant than in oxaliplatin-sensitive cells, and miR-125b downregulated EVA1A in oxaliplatin-resistant cells, whereas inhibition of miR-125b upregulated EVA1A in oxaliplatin-sensitive cells (Figs. 4c, d). Furthermore, Spearman rank correlation assessment showed an inverse correlation between miR-125b and EVA1A, confirming that miR-125b downregulates EVA1A in HCC patients (Fig. 4e). Althoug some patients had high miRNA and high EVA1A, we think it’s due to individual differences and the *P-*value was less than 0.05, so it had statistical significance.

### EVA1A plays a role in oxaliplatin resistance in HCC

**Fig. 5:**
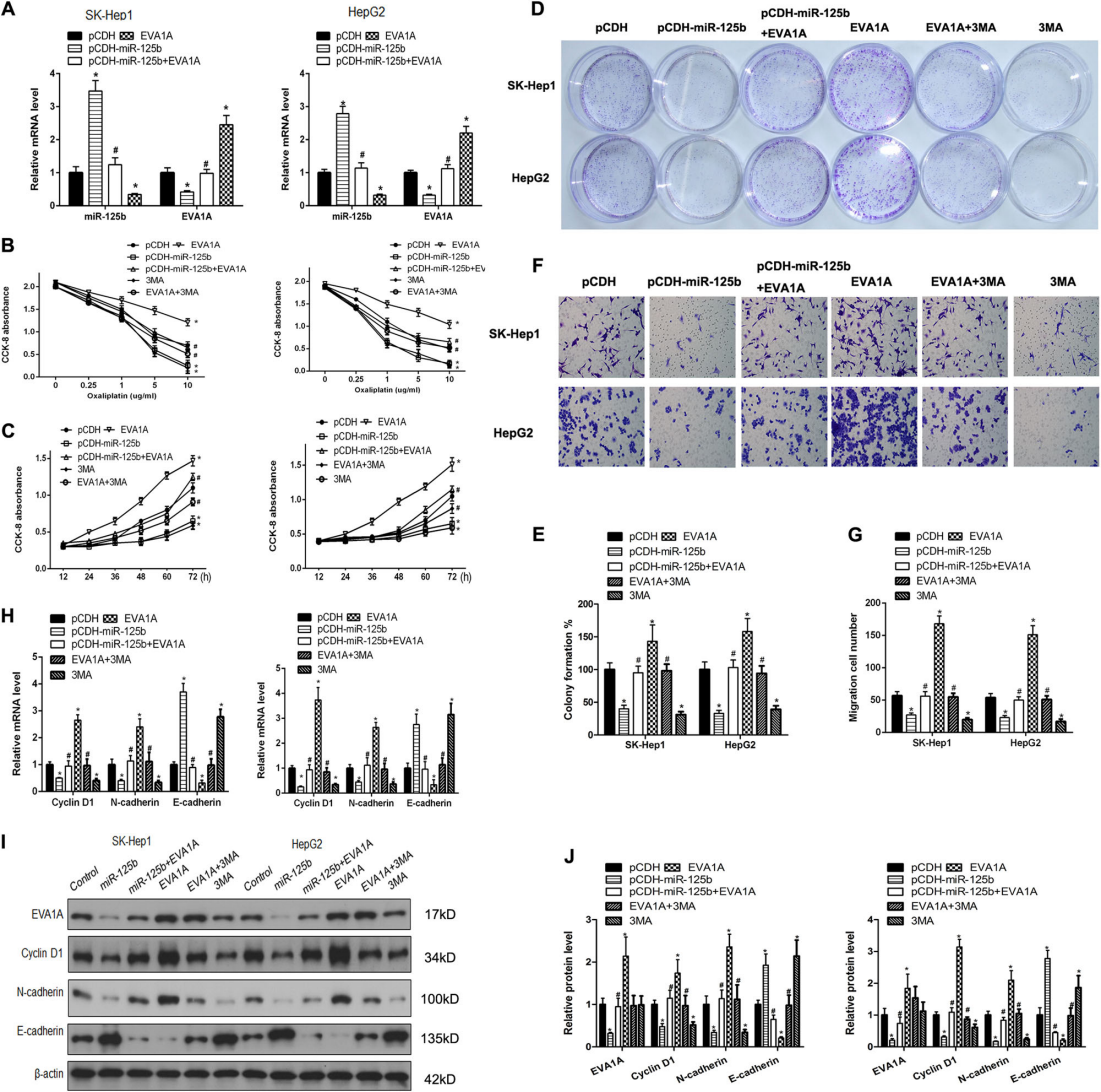
miR-125b involvement in oxaliplatin resistance is mediated by the modulation of EVA1A. **a** The expression of EVA1A and miR-125b was determined by real-time PCR treated with oxaliplatin. **b** Viability of oxaliplatin-resistant (SK-Hep1 and HepG2) cell lines ectopically expressing miR-125b and EVA1A or pretreated with 3MA treatment (5 mM) for 1 h in response to different concentrations of oxaliplatin measured using the CCK-8 assay. **c** Cell viability was determined by a CCK-8 assay in SK-Hep1 and HepG2 cells ectopically expressing miR-125b and EVA1A or pretreated with 3-MA treatment (5 mM) for 1 h treated with oxaliplatin. **d**, **e** Colony number was assessed by colony formation assays and quantified. **f**, **g** Cell migration was assessed by Transwell assays and quantified. **h**–**j** The expression of EMT markers was determined by real-time PCR and western blotting in SK-Hep1 and HepG2 cells ectopically expressing miR-125b and EVA1A or pretreated with 3-MA (5 mM) treatment for 1 h and quantified. Data are expressed as means ± SEM (*n* = 3). *P < 0.05 compared to pCDH, #*P* < 0.05 compared to EVA1A

**Fig. 6:**
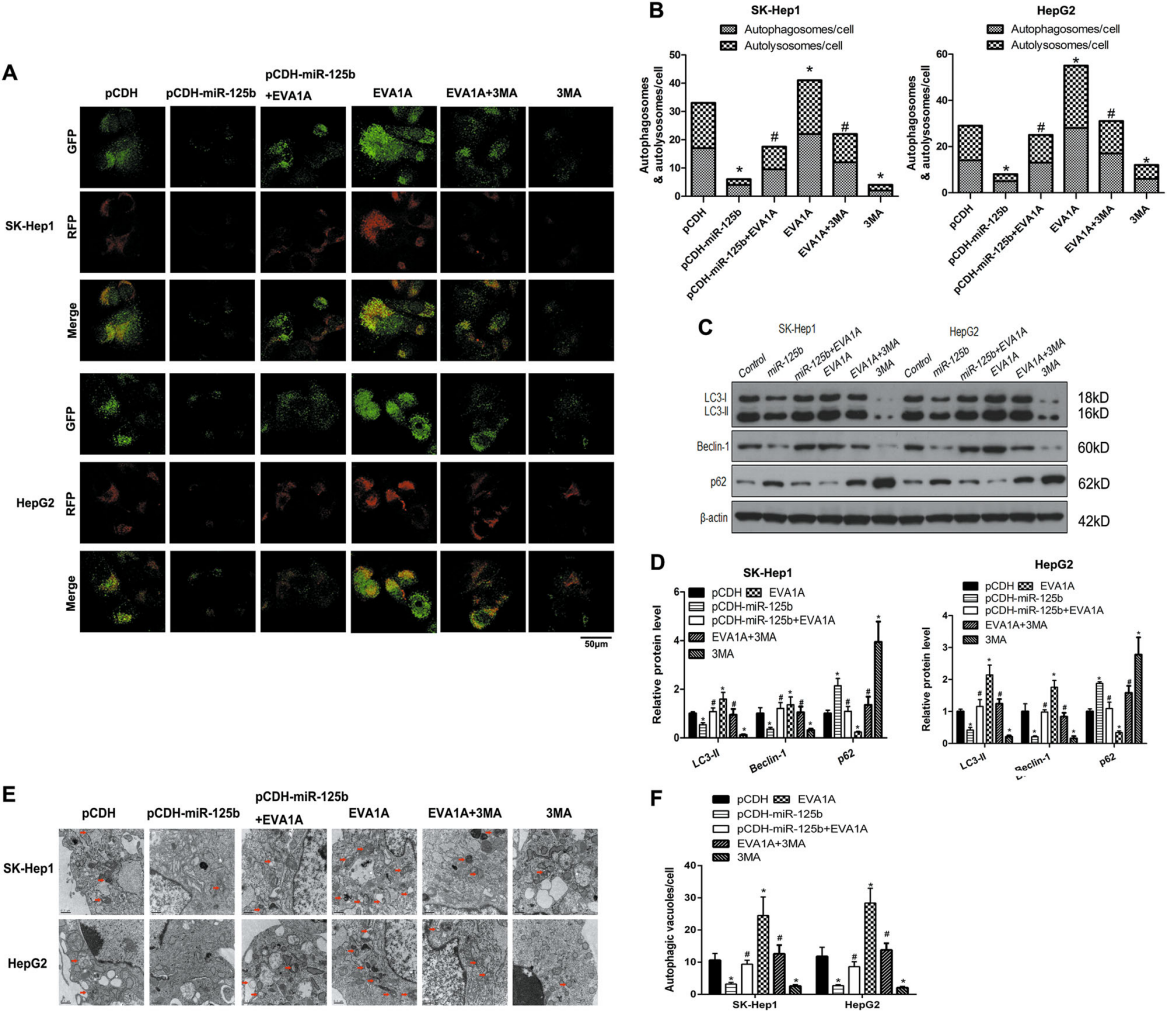
miR-125b inhibits EVA1A induced autophagy in oxaliplatin resistance. **a**, **b** Representative images of immunofluorescence staining for mRFP-GFP-LC3 in SK-Hep1 and HepG2 cells. Representative profiles of autophagosomes (RFP + GFP + dots) and autolysosomes (RFP + GFP- dots) per cell section assessed by confocal microscopy are shown and were quantified. **c**, **d** Western blotting analysis of autophagy-related proteins and quantified levels of proteins. **e**, **f** The autophagic vacuoles (autophagosomes) were detected by transmission electron microscopy (TEM). The representative TEM images are shown and the typical autophagosomes are marked with red arrows. The number of autophagosomes per cell was calculated by counting the number of double-membrane organelles in 10 cells. Data are expressed as means ± SEM (*n* = 3). *P < 0.05 compared to pCDH, #*P* < 0.05 compared to EVA1A

To investigate the involvement of EVA1A expression and the effect of miR-125b on cell proliferation and autophagy in HCC, miR-125b was coexpressed with EVA1A in SK-Hep1 and HepG2 cell lines. Real-time PCR assessment of EVA1A expression in SK-Hep1 and HepG2 cell lines showed that miR-125b downregulated EVA1A while EVA1A downregulated miR-125b expression (Fig. 5a). Cell viability decreased in a dose-dependent manner in SK-Hep1 and HepG2 cell lines, with a significantly greater decrease in viability when miR-125b was overexpressed while EVA1A overexpression reversed this decrease (Fig. 5b). Cell viability was assessed using the CCK-8 method in oxaliplatin-resistant cells treated with oxaliplatin (5 μg/ml) in which miR-125b, EVA1A or both were overexpressed using the lentivirus pCDH vector. The results showed that overexpression of miR-125b reversed the increase in cell proliferation induced by EVA1A in the SK-Hep1 and HepG2 cell lines (Fig. 5c). Consistently, overexpression of EVA1A restored the increased proliferation and migration in SK-Hep1 and HepG2 cells that were inhibited by miR-125b (Figs. 5d, e). Ectopic expression of miR-125b downregulated the EMT marker N-cadherin and the G1/S transition marker cyclin D1 and upregulated the epithelial marker E-cadherin at mRNA and protein levels, whereas co-expression of EVA1A restored expression to almost control levels, suggesting that the effect of miR-125b on suppressing EMT in HCC cells was reversed by its modulation of EVA1A expression (Figs. 5f, g). Western blotting showed that miR-125b reversed the EVA1A-induced increase in the LC3-II/LC3-1 ratio, the upregulation of Beclin-1 and downregulation of p62, suggested that miR-125b inhibited EVA1A-induced autophagy This was confirmed by immunofluorescence staining for LC3, which showed that miR-125b overexpression abolished the EVA1A-induced upregulation of LC3, indicating the formation of autophagosomes (Figs. 5h, i). Immunofluorescence staining and confocal microscopy were used to assess levels of autolysosomes and autophagosomes in HCC cells (Figs. 6a, b). Levels of lysosomes and phagosomes were reduced in cells that expressed both miR-125b and EVA1A. Western blot analysis of autophagy-related proteins and transmission electron microscopy of autophagic vacuoles confirmed these results (Figs. 6c, f). The number of autophagic vacuoles was reduced in cells cotransfected with miR-125b and EVA1A. Overall, these results indicate that miR-125b inhibits EVA1A-induced autophagy in oxaliplatin-resistant cells.

### EVA1A reverses the effect of miR-125b on tumor growth after oxaliplatin treatment in vivo

**Fig. 7:**
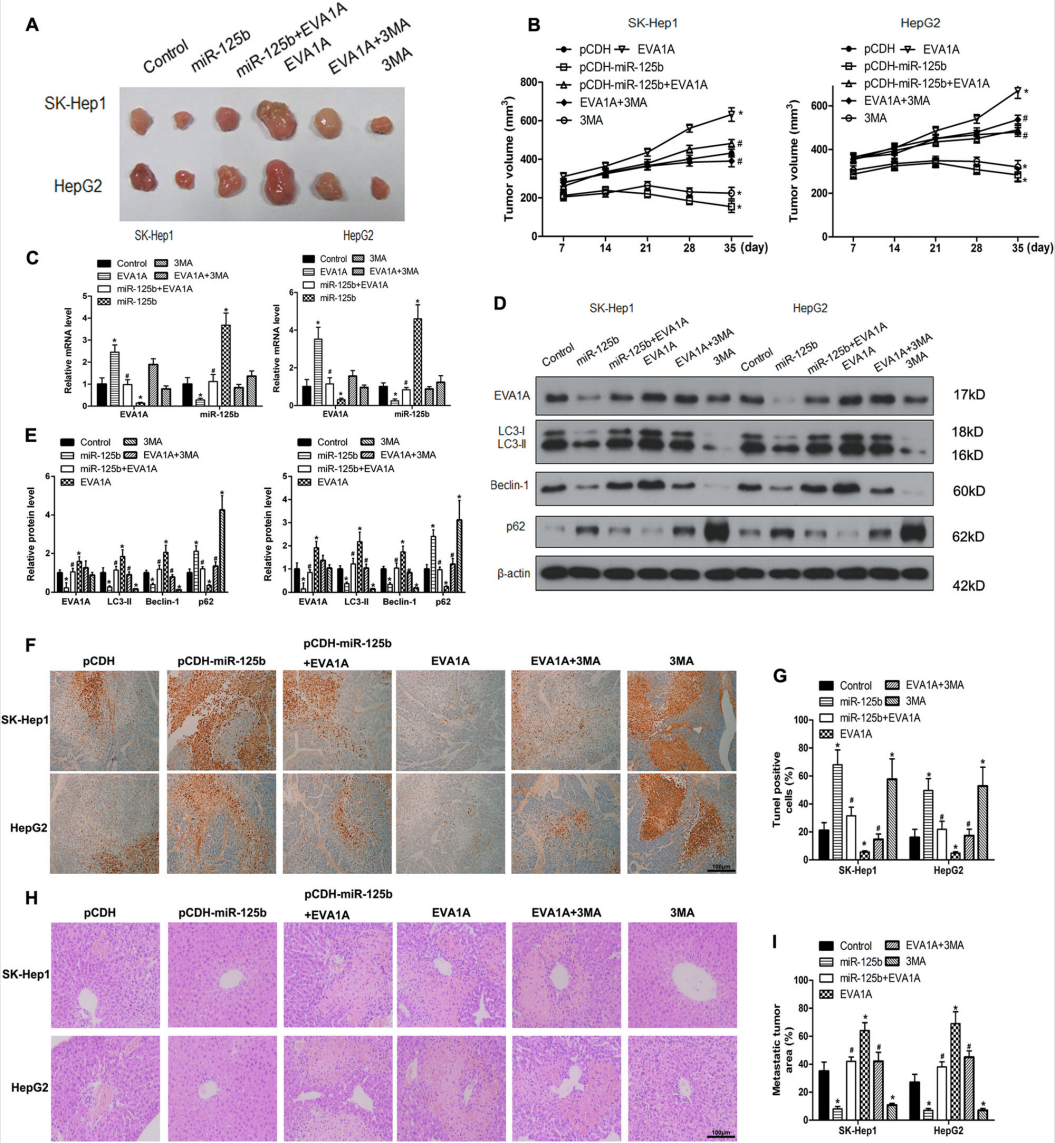
Effect of miR-125b and EVA1A in a tumor xenograft model. HCC cells stably overexpressing miR-125b or EVA1A or 3-MA treatment and treated by oxaliplatin were inoculated subcutaneously into the right flank regions of 4-week-old male BALB/c nude mice. **a** Representative images of xenograft tumors isolated from nude mice in the different groups. **b** Tumor size in the different groups. **c** The expression of EVA1A and miR-125b were detected by western blotting in the indicated groups of tumors. **d**, **e** Western blot analysis of autophagy-related proteins and quantified levels of proteins. **f**, **g** The TUNEL assay (×400) was performed to determine the apoptotic indices. **h**, **i** HCC cells (2 × 10^6^) stably expressing miR-125b or EVA1A were injected into the spleen of BALB/c nude mice under anesthesia. Mice were euthanized 8 weeks after injection, the livers were surgically excised and subjected to H&E staining to detect metastatic liver lesions. Data are expressed as means ± SEM (*n* = 6). *P < 0.05 compared to pCDH, #*P* < 0.05 compared to EVA1A

The effects of miR-125b and EVA1A on tumor growth in vivo were assessed in a mouse tumor xenograft model generated by injection of transfected SK-Hep1 or HepG2 HCC cells. The results showed that miR-125b suppressed tumor growth, whereas concomitant overexpression of EVA1A reversed the effect of miR-125b, restoring tumor size to control levels after oxaliplatin treatment (Figs. 7a, b). Real-time PCR assessment of EVA1A expression in tumors showed that miR-125b downregulated EVA1A and partially inhibited the effect of EVA1A overexpression (Fig. 7c). Western blot analysis showed that miR-125b suppressed the LC3-II/LC3-1 ratio while EVA1A reversed this suppression, and the upregulation of Beclin-1 and downregulation of p62, suggesting that miR-125b inhibited autophagy in tumors (Fig. 7d). We assessed levels of apoptosis in tumor tissue by terminal deoxynucleotidyl transferase (TdT)-mediated dUTP digoxigenin nick-end labeling (TUNEL) assay (Fig. 7e). Tissue samples from tumors transfected with miR-125b contained a higher level and EVA1A and a lower level of apoptotic cells. Assessment of liver metastasis showed that overexpression of miR-125b suppressed liver metastatic foci induced by EVA1A injection of overexpressing cells (Fig. 7f). Taken together, these results indicated that EVA1A reversed the tumor-inhibiting effect of miR-125b in xenograft tumors from oxaliplatin-resistant cells and promoted metastasis, confirming that miR-125b plays a role in preventing oxaliplatin resistance by downregulating its target EVA1A.

## Discussion

The development of resistance is one of the most important factors limiting the efficacy of chemotherapy agents such as oxaliplatin, a platinum compound that is often used as a component of combination therapy for different cancers. Oxaliplatin induces the formation of intrastrand guanine–guanine and guanine–adenine DNA links, cell cycle arrest, and cell death^13,14^. Resistance to oxaliplatin involves many mechanisms, including detoxification via the antioxidant glutathione system, activation of DNA repair systems, and the induction of antiapoptotic pathways^15–18^. Understanding the mechanisms of resistance to specific chemotherapeutic agents may help identify targets and develop more effective treatment strategies. Here, we investigated the roles of miR-125b and its target EVA1A in oxaliplatin resistance in HCC. We used two oxaliplatin-resistant and two oxaliplatin-sensitive cell lines and showed that miR-125b was downregulated in association with oxaliplatin resistance. Overexpression of miR-125b in oxaliplatin-resistant HCC cells inhibited proliferation, invasion, and EMT, suggesting that miR-125b may increase cell sensitivity to oxaliplatin. The miR-125 family is the human homolog of the miRNA lin-4, which was the first miRNA described for its role in *Caenorhabditis elegans* in 1993^19^. Members of the highly conserved miR-125 family have been implicated in many cellular processes including differentiation and metabolism, and aberrant expression of miR-125 is associated with cell proliferation, apoptosis, invasion, metastasis and immune responses in cancer cells. miR-125 plays a tumor suppressor role and is downregulated in chronic lymphocytic leukemia, HCC, melanoma, Ewing’s sarcoma, head and neck cancer, bladder cancer, and gastric cancer whereas it promotes carcinogenesis in myeloid leukemia, B-cell leukemia, non-small cell lung cancer, glioblastoma, clear-cell renal carcinoma, prostate cancer, pancreatic cancer, and oligodendroglioma^20^. In breast cancer, miR-125b was shown to be downregulated in relation to survival; however, it was also found to be upregulated and induce breast cancer cell metastasis by activating the Rho GTPase activating protein STARD13^21,22^. The role of miR-125b in the regulation of tumor cell apoptosis has been studied extensively and shown to be related to the modulation of p53 signaling networks and the downregulation of antiapoptotic proteins^20,23^. In addition, miR-125b has been implicated in the modulation of EMT via different targets including transforming growth factor β^20^. miR-125b was also shown to be involved in resistance to chemotherapy, as shown by its downregulation in doxorubicin-resistant breast cancer cells. In gastrointestinal cancer, miR-125b is upregulated in imatinib-resistant tumors, where it decreases the cytotoxicity of imatinib by downregulating STARD13 and PTPN18^24,25^. In liver cancer, miR-125b was shown to be downregulated in approximately 70% of primary HCCs, showing the ability to inhibit cell proliferation, cell cycle progression, and metastasis by targeting LIN28B^10^. The results of the present study indicate that the downregulation of miR-125b is associated with resistance to oxaliplatin treatment in HCC, which is mediated by its target EVA1A. Somatotropin is one of significantly dysregulated proteins in Eva1a^−/−^ mouse model^26^. Keane J et al. reported that the expression of miR-125b in mitochondrial fractions showed a significant down-regulation eight days post-rhGH administration^27^. We can suspicion that EVA1A regulates miR-125b through growth hormone, which requires further experimental verification.

We identified EVA1A as a target of miR-125b and showed that this autophagy-related protein was upregulated in HCC tissues from oxaliplatin-resistant patients, suggesting that it plays a role in resistance to chemotherapy. The downregulation of EVA1A by miR-125b and its effect on cell proliferation, migration, and EMT indicated a potential mechanism underlying the involvement of miR-125b in oxaliplatin resistance mediated by the modulation of its target EVA1A. EVA1A is a transmembrane protein and its overexpression induces cell autophagy, whereas its silencing inhibits starvation-induced autophagy in HeLa cells^12^. Adenovirus-mediated expression of EVA1A in various tumor cell lines promoted autophagy via inhibition of mTOR and induced apoptosis via activation of caspase-3, inhibiting tumor cell growth^28^. Autophagy is often upregulated in tumor cells in response to chemotherapy, and autophagy inhibition sensitizes tumor cells to anticancer agents. Defects in apoptosis have been linked to resistance to platinum derivatives, and impaired mitochondrial apoptosis is associated with oxaliplatin resistance in colon cancer^17^. Oxaliplatin resistance was shown to be associated with the induction of EMT in colorectal cancer cell lines, suggesting that the switch from a proliferative to an invasive phenotype is a survival mechanism contributing to chemoresistance^29^. For instance, oxaliplatin resistance may be promoted in HCC by the BMP4-mediated induction of EMT via the MEK1/ERK/ELK1 signaling pathway^30^. Moreover, as with EVA1A, the chemoresistance associated with BMP4 is believed to involve autophagy and the inhibition of apoptosis^31^. These studies support the role of autophagy and EMT in oxaliplatin resistance and provide a potential explanation for the present results, suggesting that the downregulation of EVA1A by miR-125b could inhibit autophagy and EMT, thereby contributing to the reversal of oxaliplatin resistance in HCC. Furthermore, our results in vivo, show that miR-125b overexpression reversed the growth-promoting effect of EVA1A on oxaliplatin-resistant xenograft tumors, and support the involvement of miR-125b in preventing chemoresistance by the downregulation of EVA1A and thus the promotion of cell death and reduction of autophagy in response to oxaliplatin.

## Conclusion

The results of the present study indicate that miR-125b inhibits the resistance of HCC cells to oxaliplatin via a mechanism involving the downregulation of EVA1A-mediated autophagy. The present findings suggest potential approaches of overcoming chemotherapy resistance by modulating the expression of miR-125b or its target EVA1A.

## Materials and methods

### Clinical specimens and cell lines

HCC patients who underwent FOLFOX4 chemotherapy (OXA 85 mg/m^2^ i.v. on day 1; LV 200 mg/m^2^ i.v. 2 h on days 1 and 2; and FU 400 mg/m^2^ i.v. bolus at hour 2, then 600 mg/m^2^ over 22 h on days 1 and 2, once every 2 weeks) at Tongji University Affiliated Tenth People’s Hospital were enrolled in this study. Clinical resistance to an oxaliplatin regimen was defined as the appearance of new lesions or the increase of tumor growth > 30% after 2 months of chemotherapy while the increase of tumor growth < 20% defined as non-resistance to oxaliplatin. Then we selected oxaliplatin-resistant (*n* = 38) and non-resistant (*n* = 38) HCC patients. All experiments were performed according to the Institutional Review Board approved protocol. All participants gave informed written consent before participating in this study. A surgical tumor resection was performed on each patient. Histologic sections were reviewed by two expert pathologists to verify the histologic diagnosis. All liver tissues were immediately dissected, placed on ice, snap-frozen in liquid nitrogen and stored at -80°C until processing or fixed in 4% paraformaldehyde for IHC.

The human HCC cell lines HepG2, Huh6, Mahlavu, and SK-Hep1 were obtained from Shanghai Institute of Cell Biology (Shanghai, China). Cell lines were maintained in Dulbecco’s modified Eagle’s medium supplemented with 10% fetal bovine serum (Hyclone), 1% penicillin–streptomycin amphotericin B solution, 1% l-glutamate, and 1% nonessential amino acids in a 37 °C incubator containing 5% CO_2_. All of the reagents mentioned above were obtained from Gibco (Gaithersburg, MD, USA) and Biological Industries (Beit Haemek, Israel).

### Immunohistochemistry

Thawed samples were fixed in 4% formalin and embedded in paraffin for histopathological analysis. Samples were deparaffinized with xylol and then sliced into 4-µm sections. Sections were rehydrated using a graded ethanol series. A heat-induced epitope protocol was used for antigen-retrieval (95°C for 40 min). Samples were incubated in methanol containing 0.3% hydrogen peroxide to block endogenous peroxidase. Samples were blocked with protein serum (Vectastain Elite ABC kit; Vector Laboratories, Inc., Burlingame, CA, USA) and then incubated (overnight at 4 °C) with polyclonal rabbit anti-human EVA1A antibody at 1:1000 (Abcam, Shanghai, China). After washing three times in TBST (150 mM NaCl, 10 mM Tris-HCl, pH 7.6), sections were incubated with secondary antibody for 20 min at room temperature. Peroxidase-conjugated biotin-streptavidin complex (Dako, Glostrup, Denmark) was then applied to the sections for 20 min. Sections were visualized with 3, 3′-diaminobenzidine and counterstained with hematoxylin. The negative control used nonimmune serum instead of primary antibody.

### Plasmid construction

The human miR-125b precursor and human EVA1A coding sequence were cloned into the mammalian expression vector pcDNA3.1( + ) (Invitrogen) at sites KpnI and XhoI (TaKaRa, Dalian, China) to generate stably-transfected HCC cell lines. Virus was produced in 293 T cells cotransfected with lentiviral vector pCDH and packaging plasmid.

### Cell transfection

Oligonucleotides, including miR-125b mimic and the miR-125b inhibitor anti-miR-125b, were used (Thermo Scientific, Lafayette, CO, USA) for overexpression or inhibition of miR-125b, respectively.

### RNA extraction and real-time PCR

Total RNA was extracted from tissues and cells using TRIzol reagent (Invitrogen, Carlsbad, CA, USA) according to the manufacturer’s protocol. Mature miRNA was detected by reverse transcription of miRNA using a TaqMan miRNA Reverse Transcription Kit (Applied Biosystems, Foster City, CA, USA). Real-time PCR was carried out using the appropriate TaqMan miRNA assay (Applied Biosystems) and a Prism 7500 instrument (Applied Biosystems). U6 was quantified as an endogenous control. mRNA expression was detected by reverse transcription of total RNA (500 ng) into cDNA using PrimeScript reverse transcriptase and random primers (TaKaRa), followed by real-time PCR with SYBR Green (Applied Biosystems). β-actin was used as an endogenous control. Analyses were performed in triplicate and repeated at least three times. The primer sequences used are shown in Table 3.

**Table 3:** Sequences of primers for quantitative real-time PCR

Gene name	Forward primer sequence (5′ → 3′)	Reverse primer sequence (5' → 3′)
miR-125b	CGCGCTCCCTGAGACCCTAAC	TGGTGTCGTGGAGTCG
U6	CGCTTCACGAATTTGCGTGTCAT	GCTTCGGCAGCACATATACTAAAAT
EVA1A	GCCGCTCTGTACTTTGTC	TCTCCCTGATGATTCGTT
Cyclin D1	GCGAGGAACAGAAGTGC	GAGTTGTCGGTGTAGATGC
E-cadherin	TACACTGCCCAGGAGCCAGA	TAATCCGGACACTGGTGCCA
N-cadherin	CTCCTATGAGTGGAACAGGAACG	TTGGATCAATGCATAATCAAGTGCTGTA
β-actin	CTCCATCCTGGCCTCGCTGT	GCTGTCACCTTCACCGTTCC

### Western blot analysis

Cells were directly lysed with RIPA containing protease and phosphatase inhibitors (Roche Applied Science) and proteins were separated by 10% SDS-PAGE after denaturation. Immunoblot analysis was performed by initial transfer of proteins onto polyvinylidene fluoride membranes using Mini Trans-Blot (Bio-Rad Laboratories, Richmond, VA, USA) and followed by a blocking step with 5% nonfat dried milk plus 0.1% Tween 20 for 2 h at room temperature. Membranes were immunoblotted with antibodies diluted 1000-fold overnight at 4 °C and visualized with Horse Radish Peroxidase diluted 5000-fold for 1 h at room temperature. The primary antibodies against EVA1A, Cyclin D1, N-cadherin, E-cadherin, Beclin-1, Bcl-2, LC3, and β-actin were from Cell Signaling Technology (Danvers, MA, USA). Signals were detected by FluorChem E system (Alpha Innotech Corp, Santa Clara, CA, USA).

### Cell proliferation assays

Cell proliferation was determined using a CCK-8 (Dojindo Laboratories, Kumamoto, Japan). Briefly, HCC cells were seeded into 96-well plates at an initial density of 5,000 cells per well. Cells transfected with anti-miR-125b were plated at day 1 after transfection. Ten microliters of the kit reagent were added to each well at 12, 24, 36, 48, 60, or 72 h after seeding, and all plates were scanned by a microplate reader (Thermo Scientific) after a further 2 h. Cell proliferation was evaluated by absorbance at 450 nm.

### Cell migration assays

Cell migration was analyzed using a Transwell Permeable Supports system (Corning Incorporated, Corning, NY, USA). Cells were plated in uncoated 24-well inserts with a pore size of 8 μm (BD Bioscience, Bedford, MA, USA). Cells were seeded in the upper chamber at a density of 2 × 10^4^ cells/well in serum-free medium, and serum containing 10% FBS was added to the lower chamber as a chemoattractant. Cells transfected with anti-miR-125b were plated at day 2 after transfection. Medium containing 10% FBS was added to the lower chamber, and cells were cultured for 12 h at 37 °C. Subsequently, non-migrating cells in the upper chamber were gently removed with a cotton swab and wells were washed, stained with 0.1% Crystal violet and analyzed by inverted microscopy. The migrating cells were counted in five randomly-selected areas to evaluate the migration activity of the indicated cells.

### Colony formation assay

Cell proliferation was assessed by a soft agar colony formation assay. A 6-well plate containing a 1.5 ml bottom layer and 0.5 ml top layer of agar was used (5.1 mg/mL, Difco Laboratories, Detroit, MI, USA). Cells (10^4^/well) were transferred onto the bottom layer and then overlaid with a top layer and cultured at 37 °C in 5% CO_2_. Giemsa staining was used to quantify the formation of colonies on the seventh day.

### Luciferase reporter assays

Luciferase reporter assays were conducted using the Dual-Luciferase Reporter Assay System (psiCHECK-2 vector; Promega, Madison, WI, USA). A fragment of the EVA1A 3′ UTR containing either the predicted binding site for miR-125b or a mutated or deleted 3′ UTR was inserted into the psiCHECK2 vector. After verification by DNA sequencing, the psiCHECK-2 vector containing either the wt, mutated, or deleted EVA1A 3′ UTR was transfected into HCC cells with or without synthetic miR-125b mimic, using a Lipofectamine RNAiMAX kit (Invitrogen) following the manufacturer’s instructions. At 36 h after transfection, luciferase activity was detected using a dual-luciferase reporter assay system and normalized to Renilla activity. Data were normalized to the luciferase activity of cells transfection with miR-control.

### Autophagic flux analysis

mRFP-GFP-LC3-HCC cells were fixed with 4% paraformaldehyde (PFA, Sigma-Aldrich) and stained with 10 μM Hoechst33342 (Sigma-Aldrich) after treatment with 3-MA (5 mM) for 1 h. Images of the cells were obtained from the Operetta High Content Imaging System (Perkin-Elmer) and analyzed using Harmony Analysis Software (Perkin-Elmer). Cells were detected with green (GFP) or red (mRFP) fluorescence. Autophagosomes are yellow puncta and autolysosomes are only red puncta in merged images. Autophagic flux was determined by the increased percentage of only red puncta in the merged images.

### Electron microscopy

Cells were fixed with 2.5% glutaraldehyde in phosphate buffer and stored at 4 °C until embedding. Cells were postfixed with 1% osmium tetroxide followed by an increasing gradient dehydration step using ethanol and acetone. Cells were then embedded in Araldite, and ultrathin sections were obtained (50–60 nm), placed on uncoated copper grids, and stained with 3% lead citrate–uranyl acetate. Images were examined with a CM-120 electron microscope (Philips).

### Tumorigenesis in nude mice

Male BALB/c nude mice (4 weeks of age) were purchased from the Shanghai Laboratory Animal Center of the Chinese Academy of Sciences (Shanghai, China; *n* = 6 per group). HCC cells (1 × 10^6^) stably expressing miR-125b or EVA1A were collected and inoculated subcutaneously into the right flank regions of mice. After 7 days, mice were injected intraperitoneally with oxaliplatin (5 mg/kg) and vehicle or 3-MA (25 mg/kg) every day. Tumor nodules were measured every 7 days using calipers. Mice were euthanized after 1 month and the tumor growth rate and rate of inhibition were calculated. All animal studies were approved by the Ethics Committee of Shanghai 10th People’s Hospital. All mice were housed in specific pathogen-free conditions following the guidelines of the Institutional Animal Care and Use Committee of Tongji University School of Medicine.

### Animal model of liver metastasis

The effect of miR-125b and EVA1A expression on HCC metastasis was determined in xenograft mouse models of liver metastasis. Nude male BALB/cA-nu mice (6-weeks-old) were obtained from Shanghai SLAC Laboratory Animal Co., Ltd. (Shanghai, China; *n* = 6 per group). In the liver metastasis model, the indicated cells were suspended as single cells (2 × 10^6^) in 100 μL phosphate-buffered saline and were slowly injected into the spleen of BALB/c nude mice under anesthesia. After 7 days, mice were injected intraperitoneally with oxaliplatin (5 mg/kg) and vehicle or 3-MA (25 mg/kg) every day. Mice were euthanized 8 weeks post-injection; the livers were surgically excised and subjected to hematoxylin and eosin staining. The metastatic lesion was observed under a microscope. All animal studies were approved by the Ethics Committee of Shanghai 10th People’s Hospital. All mice were housed in specific pathogen-free conditions following the guidelines of the Institutional Animal Care and Use Committee of Tongji University School of Medicine.

### TUNEL assay

For apoptosis quantification, HCC xenograft sections were processed for in situ immunocytochemical localization of nuclei exhibiting DNA fragmentation by the TUNEL technique using an apoptosis detection kit (In Situ Cell Death Detection Kit, POD, Roche, 11684817910). The protocols were followed according to the manufacturer’s instructions. Paraffin-embedded samples (3-µm-thick sections) were deparaffinized and rehydrated with xylene and ethanol and permeabilized with 20 µg/ml of proteinase K (Gibco, Grand Island, NY, USA). Endogenous peroxidase was inactivated by coating the samples with 3% H_2_O_2_. Sections were rinsed with PBS and then immersed for 60 min in TdT buffer at 37 °C and incubated for 30 min with antidigoxigenin peroxidase conjugate, followed by peroxidase substrate (3′-diaminobenzidine tetrahydrochloride [DAB]). Finally, sections were counterstained with 0.5% (wt/vol) methyl green.

### Statistical analysis

Statistical analyses were performed using SPSS version 13.0 (SPSS Inc., Chicago, IL, USA). Values were presented as the mean ± SD from three independent experiments. Differences/correlations between groups were calculated using Student’s *t*-tests or Spearman’s correlation analysis. A *P*-value < 0.05 was defined as significant.
