# Correction to: MicroRNA-125b reverses oxaliplatin resistance in hepatocellular carcinoma by negatively regulating EVA1A mediated autophagy

**DOI:** 10.1038/s41419-018-0836-y

**Published:** 2018-08-24

**Authors:** Wei-Wei Ren, Dan-Dan Li, Xiaolan Chen, Xiao-Long Li, Ya-Ping He, Le-Hang Guo, Lin-Na Liu, Li-Ping Sun, Xiao-Ping Zhang

**Affiliations:** 1 Department of Medical Ultrasound, Shanghai Tenth People’s Hospital, Ultrasound Research and Educational Institute, Tongji University School of Medicine, 200072 Shanghai, China; 2 Department of Radiotherapy, Shanghai Tenth People’s Hospital, Ultrasound Research and Educational Institute, Tongji University School of Medicine, 200072 Shanghai, China; 3 Department of Interventional & Vascular Surgery, Tongji University School of Medicine, 200072 Shanghai, China

**Correction to**: *Cell Death and Disease* (2018) **9**:547; 10.1038/s41419-018-0592-z; published online 10th August 2017.

Following publication of the article, the authors reported that Huixiong Xu had left the department, and requested that the author list be amended accordingly.

The PDF and HTML versions of the paper have been modified accordingly.

